# MS-*k*NN: protein function prediction by integrating multiple data sources

**DOI:** 10.1186/1471-2105-14-S3-S8

**Published:** 2013-02-28

**Authors:** Liang Lan, Nemanja Djuric, Yuhong Guo, Slobodan Vucetic

**Affiliations:** 1 Department of Computer and Information Sciences, Temple University, Philadelphia, PA, 19122, USA

## Abstract

**Background:**

Protein function determination is a key challenge in the post-genomic era. Experimental determination of protein functions is accurate, but time-consuming and resource-intensive. A cost-effective alternative is to use the known information about sequence, structure, and functional properties of genes and proteins to predict functions using statistical methods. In this paper, we describe the Multi-Source *k*-Nearest Neighbor (MS-*k*NN) algorithm for function prediction, which finds *k*-nearest neighbors of a query protein based on different types of similarity measures and predicts its function by weighted averaging of its neighbors' functions. Specifically, we used 3 data sources to calculate the similarity scores: sequence similarity, protein-protein interactions, and gene expressions.

**Results:**

We report the results in the context of 2011 Critical Assessment of Function Annotation (CAFA). Prior to CAFA submission deadline, we evaluated our algorithm on 1,302 human test proteins that were represented in all 3 data sources. Using only the sequence similarity information, MS-*k*NN had term-based Area Under the Curve (AUC) accuracy of Gene Ontology (GO) molecular function predictions of 0.728 when 7,412 human training proteins were used, and 0.819 when 35,622 training proteins from multiple eukaryotic and prokaryotic organisms were used. By aggregating predictions from all three sources, the AUC was further improved to 0.848. Similar result was observed on prediction of GO biological processes. Testing on 595 proteins that were annotated after the CAFA submission deadline showed that overall MS-*k*NN accuracy was higher than that of baseline algorithms Gotcha and BLAST, which were based solely on sequence similarity information. Since only 10 of the 595 proteins were represented by all 3 data sources, and 66 by two data sources, the difference between 3-source and one-source MS-*k*NN was rather small.

**Conclusions:**

Based on our results, we have several useful insights: (1) the *k*-nearest neighbor algorithm is an efficient and effective model for protein function prediction; (2) it is beneficial to transfer functions across a wide range of organisms; (3) it is helpful to integrate multiple sources of protein information.

## Background

Determining biological functions of proteins is a key challenge in the post-genomic era. The experimental methods for protein function prediction are time-consuming and resource-intensive. It is infeasible to experimentally determine the functions of all known proteins. For that reason, computational methods that predict biological functions of a protein using known information about its sequence, structure, and functional behaviour, are becoming an attractive low-cost alternative. During the recent couple of decades, many computational methods for predicting protein function have been developed, and the 2011 Critical Assessment of Function Annotations (CAFA) has been designed to establish a state of the art in the field.

The sequence alignment-based function inference is the most widely used form of computational function prediction [1]. These approaches use sequence comparison tools, such as BLAST [2], to search annotated databases for the most similar proteins to the query protein based on sequence and transfer their functions. The biological rationale for sequence comparison is that if two sequences are similar, then they probably evolved from a common ancestor and have similar functions. Gotcha [3] is a similar method that takes sequence alignment scores between a query protein and a database of functionally annotated proteins, and overlays them on functional ontology, cumulatively propagating the scores towards the root of the ontology. Both the BLAST and Gotcha approaches were used as baselines in 2011 CAFA.

Beyond sequence similarity, several computational approaches have been proposed to utilize other types of biological data, such as protein-protein interactions (PPI) and gene expression data. The methods that use PPI data to predict protein functions are based on a simple premise: a protein does not perform its function in isolation; instead, a group of proteins needs to interact in order to perform a certain function. Therefore, the functions of a querying protein can be inferred from its interacting partners. Schwikowski et al. [4] used a neighbor counting method, where a function is assigned to the querying protein based on the number of its neighbors in the PPI graph which have this function. Hishigaki et al. [5] extended this method, by considering proteins that could be reached via *n *links, instead of considering only the direct neighbors.

Use of gene expression data for function prediction has been motivated by an observation that co-expressed genes are likely to be functionally related [6-8]. In the seminal work by Eisen et al., [7] based on the co-expression data, genes were clustered into a number of groups and the functions transferred to all genes in a cluster. Machine learning-based approaches where function prediction is studied as a multi-label classification problem have also been popular. There, a function is predicted from gene expression measurements across several microarrays. For example, in an early work of this type, Brown et al. [9] applied Support Vector Machines classifier [10] to the task of learning functions from yeast gene expression data.

Arguably, each data source captures only one aspect about proteins' properties. Thus, combining such heterogeneous data can bring a more complete picture about protein function. Recently, several studies showed promising improvements in protein function prediction by integrating multiple types of biological data. Troyanskaya et al. [11] proposed a Bayesian network model to infer the posterior probability functional linkage between two genes given their functional relationship observed from multiple data sources. Barutcuoglu et al. [12] integrated different data sources by concatenating all feature vectors from different data sources for a protein into a single feature vector. Mostafavi and Morris [13] assigned weights to different data sources by solving a constrained linear regression problem, which minimized the least square error between the composite network and the target network constructed from the label vector, on sets of related functional categories. Despite these and related efforts, how to effectively integrate different types of biological data for protein function prediction remains a largely open question.

There are several challenges that need to be addressed in future research on multi-source function prediction. The first is that different sources of information may have vastly different coverage. For example, while sequence similarity covers all known proteins, PPI data coverage is significantly smaller, and gene expression similarities are constrained by a specific microarray platform. The second challenge is differences in data quality. For example, PPI can be obtained by a variety of techniques that differ in cost and reliability. A confounding issue is that functional annotations have an uneven coverage biased towards certain types of proteins and functions, and that determination of protein functions, such as the one provided by Gene Ontology [14], is a subjective and error-prone process.

In an attempt to address some of the identified challenges and faced with the tight deadline of 2011 CAFA, we focused our attention on the *k*-nearest neighbor approach for function prediction proposed in [15]. This is an easy to implement, intuitive, and relatively fast algorithm that searches for *k *nearest neighbors of the query sequence and transfers their functions by weighted averaging, such that nearer neighbors have larger influence to prediction than the farther ones. In this paper, we propose the Multi-Source *k*NN (MS-*k*NN) algorithm able to use multiple sources of protein information. To provide the final prediction, MS-*k*NN uses weighted averaging of the source-specific prediction scores. In the algorithm design, we explored several approaches to determine weights, ranging from averaging to solving a constrained optimization problem. We observe that a query protein does not have to be present in all data sources. For example, we might know the protein's sequence and whether it interacts with other proteins, but not its gene expression (e.g., because its gene is not printed on a microarray, or because microarray data are not available for the host organism). Averaging of the source-specific scores provides a simple mechanism for dealing with potential missing predictions.

In the following, we will discuss evaluation of several prediction approaches prior to CAFA submission deadline, describe how we selected the predictor, summarize and discuss results on CAFA proteins, and propose some directions for the future research.

## Results

### CAFA challenge

At the beginning of the challenge, 48,298 proteins were released by CAFA organizers as the test proteins. In the released test data, the protein names, Uniprot IDs, and sequences were provided. A large majority of these proteins did not have known functional assignments, as defined by the Gene Ontology (GO) [14] annotations in Swiss-Prot database. The organizers did not provide any training data to the participants. Therefore, the participants were free to use any available information about proteins and genes they found suitable. The objective of the assessment was to predict functions of the test proteins. The success was measured by evaluating the prediction accuracy of GO annotations of the test proteins that became available after the submission deadline.

### Data sources

**Figure 1:**
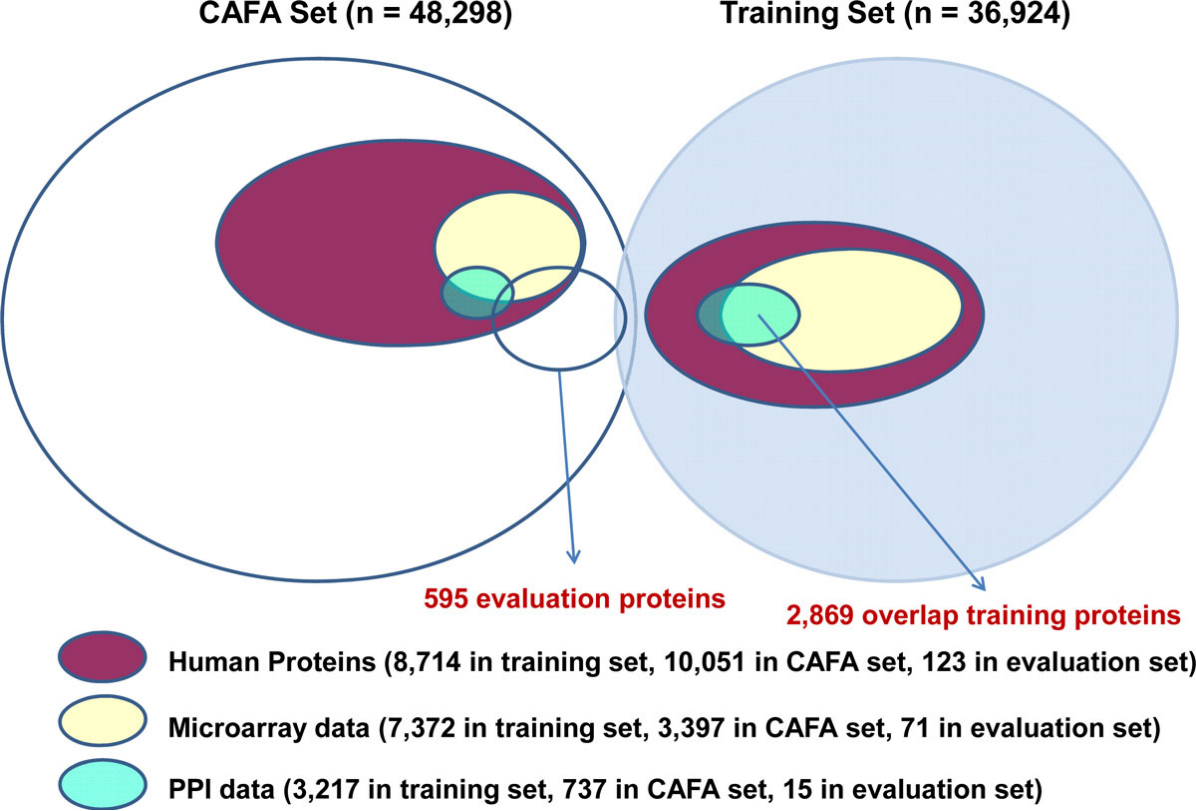
**Visual summary of the datasets**.

We considered integration of three different data sources for protein function prediction. These three data sources were: (1) protein sequence data; (2) microarray expression data; and (3) protein-protein interaction data. Particularly, prior to CAFA deadline we focused on human proteins, in order to more easily evaluate and characterize our approaches. Visual summary of the data sets we used is in Figure 1.

#### Protein sequence data

By courtesy of Dr. Predrag Radivojac from Indiana University, we obtained a data set of GO annotations of 36,924 proteins, as well as their pair-wise sequence similarities (expressed as percent identity), and pair-wise similarities between these proteins and the 48,298 CAFA proteins. These 36,924 proteins with their GO annotations and sequences were used as the training set for function prediction. We note that there were 474 proteins that were present in both training and CAFA data set, as they were already partially annotated. To simplify the experimental design, these 474 proteins were excluded from the training set during pre-CAFA evaluation. We still provided function predictions for them, as they were in the CAFA data set.

#### Microarray Expression Data

We downloaded 392 Affymetrix GPL96 Platform microarray datasets from GEO (http://www.ncbi.nlm.nih.gov/geo/). The GPL96 is one of the most widely used human microarray platforms. We linked the Affymetrix probe IDs with Uniprot IDs through Entrez. After ID mapping, the microarray data covered 7,372 human proteins in the training set, and 3,397 human proteins in CAFA set. These datasets were already pre-processed by Affymetrix Microarray Suite Version 5.0 and we did not apply any additional pre-processing.

#### Protein-protein interaction data

For PPI data source, we used physical interactions between human proteins listed in OPHID database (http://www.phenopred.org/). This data source includes 41,457 interactions between 9,141 proteins. After ID mapping, the PPI data source covered 3,217 proteins in the training set, and 737 proteins in CAFA set.

We summarize the information about each data source in Table 1.

**Table 1:** Summary of different data sources

Data source	Training size	CAFA size
Protein sequence similarity	36,924	48,298
Microarray expression	7,372	3,397
Protein-protein interaction	3,217	737

### Empirical results before CAFA

There were 2,869 annotated human proteins in the training set that were represented by all 3 data sources. Among them, we randomly selected 1,302 proteins as test set in our pre-CAFA analysis. Given the prediction scores on the 1,302 test proteins for function *f*, the True Positive Rate (TPR) and False Positive Rate (FPR) was calculated at different discrimination thresholds, creating the Receiver Operating Characteristic (ROC) curve. The Area Under the ROC Curve (AUC) was calculated by integrating the ROC curve, which is corresponded to the TermAUC evaluation metric of CAFA. We only considered the GO functions having more than 15 annotated proteins among the 1,567 3-source human proteins left after removing the 1,302 test proteins. This resulted in 122 molecular function (MF) and 546 biological process (BP) GO terms. We used *k *= 20 in all experiments in this section. In the following, we will discuss performance of several proposed function prediction algorithms.

#### Baseline vs. *lin-sim k*NN classifier

In this section, we compare the accuracies of the two different prediction algorithms: baseline *k*NN classifier and *lin-sim *incorporated *k*NN classifiers. The results for *k*NN using sequence similarity were based on the training set of 1,567 3-source human proteins, remaining after exclusion of 1,302 test proteins. While we had 392 different mi-croarray datasets available for this experiment, due to the tight deadline we used only the largest microarray data set (having 221 microarrays) with GEO accession number GSE4475.

The results in Table 2 show that the *lin-sim k*NN classifier had slightly higher accuracy than the baseline *k*NN. However, to estimate the *lin-sim *between all GO terms and to use them during prediction time is very time-consuming. This includes a need to determine the *lin-sim *function similarity threshold through cross-validation. As a result, we reasoned that the accuracy improvement was not large enough to justify use of *lin-sim k*NN predictor in CAFA.

**Table 2:** Comparison of average TermAUC of two different prediction algorithms

Data sources	122 MF terms		546 BP terms	
	***k*NN**	***lin-sim k*NN**	***k*NN**	***lin-sim k*NN**
Sequence Similarity	0.671	**0.688**	0.557	**0.558**
Microarray	0.555	**0.561**	0.563	**0.578**
PPI	0.574	**0.592**	0.580	**0.611**

#### Paralogous vs. orthologous sequences

In this section, we explore how useful it is to transfer functions from paralogous and orthologous proteins. Paralogous proteins are similar proteins within the same organism that are probably created by duplication and functional divergence. Orthologous proteins are similar proteins across different organisms that are related by speciation. The test set was still the 1,302 human proteins. We used 10 different training sets, after excluding the 1,302 test proteins: (1) 1,567 human training proteins represented by all 3 sources (as in Table 2); (2) 7,412 human proteins in training data; (3) all 16,442 proteins from human, mouse, and rat in training data; (4) 16,754 proteins from all mammals in training data; (5) all 35,622 training proteins; (6) randomly selected 7,412 proteins from set (3); (7) randomly selected 7,412 proteins from set (4); (8) randomly selected 7,412 proteins from set (5); (9) randomly selected 7,412 non-human mammal proteins; (10) randomly selected 7,412 non-human proteins. The baseline *k*NN classifier was used as the prediction model and we used the same GO terms as in Table 2.

The average TermAUC accuracies for MF and BP terms are shown in Tables 3 and 4. The results for training set from (6) to (10) are averages of 5 random selections. The results in Table 3 show that TermAUC grew with the number of annotated sequences. Interestingly, it was the largest when all available proteins were used, which included evolutionary distant prokaryotes. These results could be explained by the fact that as the training set of sequences grows, it becomes more likely that truly similar sequences are found among the *k *nearest neighbors of the query sequence. In Table 4, we show Term AUC for training sets of the same size. We observe that the highest accuracy was obtained by using non-human mammal proteins. The lowest accuracies were obtained either by exclusively human training proteins (set 2) or a sample including all proteins (sets 8 and 10). This indicates that the most useful proteins for function prediction are orthologs from closely related organisms. However, by comparing Tables 3 and 4, it is evident that it is preferable to simply use all available training proteins.

**Table 3:** Average TermAUC based on 5 training sets of different size.

Training Set (Training Size)	122 MF terms	546 BP terms
	TermAUC	TermAUC
(1) HUMAN (1,567)	0.671	0.557
(2) HUMAN (7,412)	0.728	0.609
(3) HUMAN + MOUSE + RAT (16,442)	0.807	0.692
(4) All Mammals (16,754)	0.812	0.696
(5) All Organisms (35,622)	**0.819**	**0.707**

**Table 4:** Average TermAUC based on 7 training sets with same size

Training Set (Training Size)	122 MF terms	546 BP terms
	TermAUC	TermAUC
(2) HUMAN (7,412)	0.728	0.609
(6) HUMAN + MOUSE + RAT (7,412)	0.771	0.648
(7) All Mammals (7,412)	0.762	0.649
(8) All Organisms (7,412)	0.729	0.628
(9) All Mammals excluding Human(7,412)	**0.779**	**0.659**
(10) All Organisms excluding Human (7,412)	0.721	0.623

#### Integrating predictions from multiple data sources

In this section, we compare the results of single-source *k*NN and the proposed multi-source *k*NN algorithms described in the Methods section. We used the same 1,302 human test proteins for testing as in the previous two subsections. For sequence similarity data, we used training protein sequences from all organisms, because that resulted in the highest accuracy according to Tables 3 and 4. For the microarray expression data, we used all 392 Affymetrix GPL96 microarray data sets. The prediction score was calculated as the average score among the 392 microarray data sets.

The average TermAUC for single and multi-source *k*NN are shown in the Table 5. At the level of the single-source predictors, it could be seen that the prediction based on sequence similarity was much more accurate than the microarray and PPI-based predictors on both MF and BP functions. By comparing the microarray-based predictor based on 392 microarrays listed in Table 5 with the microarray predictor based on a single microarray data set listed in Table 2, it can be concluded that it was beneficial to combine predictions from multiple microarray data sets.

**Table 5:** Comparison of AUCs of different methods

Data Source	122 MF terms	546 BP terms
	TermAUC	TermAUC
*k*NN: Sequence similarity	0.819	0.707
*k*NN: PPI	0.574	0.580
*k*NN: Microarray	0.635	0.642
MS-*k*NN	**0.848**	**0.763**
MS-W-*k*NN	0.829	0.758
MS-CW-*k*NN: root level	0.831	0.715
MS-CW-*k*NN: first level	0.851	0.702

We observe that by averaging scores from all 3 data sources using MS-*k*NN TermAUC increased by 0.03 on MF and 0.06 on BP functions as compared to using sequence similarity only. This result is very interesting considering superiority *k*NN accuracy using sequence similarity as compared to the ones using PPI and microarray data. Such result clearly indicates that integration of multiple data sources can be beneficial for protein function prediction. Accuracies of the weighted versions of MS-*k*NN were not as high as its basic version. This was a somewhat unexpected result. Upon a more careful study of the optimization problem stated in (5), we concluded that the issue lies in the interpretation of zero labels. Formulation (5) assumes that *Y_ij _*= 0 means that *i*-th protein does not have *j*-th function. However, *Y_ij _*= 0 often means that the function is not known and not accounting for this results in reduced accuracy. We think that this is a valuable insight that might be helpful in design of future predictors of protein function.

It might be somewhat surprising that MS-*k*NN is able to improve prediction scores using sequence similarity with seemingly inferior prediction scores coming from PPI and microarray data. In order to understand why two seemingly inferior predictors can help the superior one, in Table 6 we show prediction scores and ranks of 7 test proteins annotated with function GO:0044106 (cellular amine metabolic process) obtained by 3 single-source predictors and by their averaging. We note that the predicted scores from each individual data source ranged from [0, 20] because we set the parameter *k *in *k*NN to 20. We can see that TermAUC obtained with sequence data (0.829) was much larger than with microarray data (0.613) and PPI data (0.564). However, integrating these 3 sources improved the TermAUC to 0.938. For the first 5 proteins listed in Table 6 (NOS1_HUMAN, NOS3_HUMAN, OAZ1_HUMAN, OAZ2_HUMAN and SYK_HUMAN), we can see that they were ranked very high by the sequence similarity-based predictor. The addition of prediction scores from other two sources resulted in a slight decrease in their rank. For the last two proteins in Table 6, the sequence similarity-based predictor gave score 0, indicating that none of their *k *= 20 nearest neighbors were annotated with GO:0044106. Microarray-based score of PEPD_HUMAN was relatively small, but it was sufficient to improve its ranking near the top one third. In case of PON1_HUMAN, it had the top ranking based on PPI data and a very high ranking based on microarray data, such that it was ranked 18^th ^in aggregate.

**Table 6:** Prediction score and rank for test proteins annotated by GO:0044106

Proteins	Microarray	PPI	Sequence	Average
	(AUC: 0.6127)	(AUC:0.5641)	(AUC: 0.8285)	(AUC: 0.9379)
SYK_HUMAN	0.14(1203)	0 (NaN)	2.17 (2)	0.77 (3)
NOS3_HUMAN	0.23 (212)	0 (NaN)	1.95 (3)	0.73 (6)
NOS1_HUMAN	0.29 (19)	0 (NaN)	1.92 (4)	0.74 (5)
OAZ2_HUMAN	0.17 (882)	0 (NaN)	1.80 (6)	0.66 (8)
OAZ1_HUMAN	0.18 (820)	0 (NaN)	1.63 (7)	0.60 (9)
PEPD_HUMAN	0.22 (340)	0 (NaN)	0 (NaN)	0.07 (544)
PON1_HUMAN	0.26 (66)	1 (3)	0 (NaN)	0.42 (18)

### CAFA results

#### Algorithm selected for CAFA

By considering the results presented above, we observed that *lin-sim k*NN classifier improves prediction performance only slightly, while it is computationally costly and sensitive to the *lin*-*sim *threshold choice. Therefore, due to the time constraints of the competition, we decided not to use the *lin-sim *approach for score calculation. We used MS-*k*NN as our predictor because, as it can be seen from Table 5, it was more accurate than single-source *k*NN and both simpler and more accurate than other MS-*k*NN algorithms we studied. A given CAFA protein could be represented in one, two or three sources. If a data source was not available for a test protein, the score for that source in MS-*k*NN was set to zero. In this way, scores of proteins represented by multiple sources were biased upwards, reflecting increased prediction confidence.

For CAFA assessment we provided predictions for all 48,298 CAFA proteins and for all GO terms (8,728 MF terms and 18,982 BP terms). One of the rules of CAFA was that, for the final submission, one protein cannot be associated with more than 1,000 GO terms. Thus, we sorted the prediction scores for each protein and submitted the top 1,000 GO terms with the corresponding prediction scores. We note that for vast majority of CAFA proteins (44,471 out of 48,298) we only had sequence information available.

#### CAFA proteins used for testing

Only 595 of the CAFA proteins were experimentally annotated after the submission deadline, and they were used to evaluate the prediction accuracy. Of these 595 proteins, 366 proteins were associated with MF functions and 436 with BP functions. In the evaluation set, there were 2,786 new MF annotations and 11,075 new BP annotations. Among the 595 proteins, only 10 were covered by all 3 data sources, while 66 were covered by 2 of the 3 data sources. For the remaining 519 proteins we only had sequence information.

#### Baseline predictors

The CAFA organizers used the following 3 baseline algorithms for comparison with the submitted predictions.

(1) **Priors**. Prediction score of every protein for a given GO term was the same and was calculated as the probability of that GO term occurring in Swissprot. This approach made it more likely for a protein to be annotated with a more common GO term.

(2) **BLAST**. To obtain prediction score for annotation of a target protein with a GO term, the protein's sequence was compared with all protein sequences annotated with this GO term using BLAST. The sequence identity of the most similar protein was used as the prediction score.

(3) **Gotcha **[3]. Using the same BLAST output as (2), Gotcha prediction score was calculated as the sum of the negative logarithms of the E-value of the alignments between the target protein and all proteins associated with the given term.

#### Evaluation measures

The CAFA organizers used 4 different evaluation methods; 3 of them were *protein-centric *and one was *function-centric*. In this paper, we report only on the AUC results for simplicity of analysis. In protein-centric evaluation methods, the prediction scores of each protein across all available GO functions are sorted. Then, AUC is calculated for each protein. In function-centric evaluation method, for each function, the prediction scores of all proteins associated with this particular function are sorted and AUC is calculated for each GO term.

##### Threshold

At each threshold, precision and recall are calculated and reported as averages across all proteins. If a particular score for a term/protein pair is above the given threshold, then the annotation at that threshold is propagated towards the root of the ontology.

##### Top N

For a particular protein, the scores are first sorted. Then, for the highest 20 scores, precision and recall are calculated. If there is a tie between more than one term, all such terms are used to calculate AUC.

##### Weighted threshold

For each threshold, weighted precision and recall are calculated based on the information content of each term. The information content of a GO term is calculated from the January 2011 version of Swissprot, as the negative log of the frequency of the term among proteins annotated with at least one experimental evidence code.

##### TermAUC

If more than 25 of the 595 test proteins were annotated by a GO term after the CAFA deadline, Term AUC was calculated. For TermAUC, precision and recall are calculated at each threshold after propagating annotations for testing proteins. We note that we used TermAUC during the design of MS-*k*NN algorithm.

#### CAFA results

**Figure 2:**
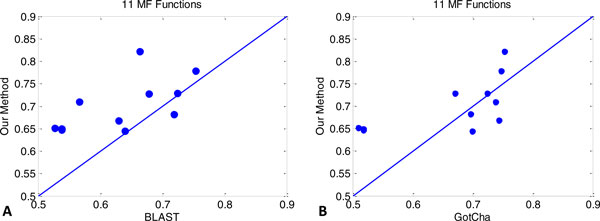
**AUC comparison on 11 MF functions**.

The AUC scores for MF terms based on different evaluation methods are shown in Table 7. The AUC scores for BP terms based on different evaluation methods are shown in Table 8. The reported TermAUC accuracies are average AUC accuracy on 11 MF and 25 BP GO terms. We provide results for sequence-based *k*NN and MS-*k*NN. The results over the 4 different AUC accuracies show that MS-*k*NN worked the best overall on MF prediction. Particularly, the improvements in Threshold and Weighted threshold AUC were quite large. For the TermAUC, sequence-based *k*NN and MS-*k*NN had similar results. This could be explained by the fact that for 519 of the 595 test proteins we used only sequence information. In BP predictions, MS-*k*NN was also overall the most accurate, although in was not the most accurate on any of the 4 accuracy measures. For a further insight, TermAUC of the 11 MF terms based on different prediction methods are compared in Figure 2. We can see that MS-*k*NN was better than BLAST on all but one MF term. It was better than Gotcha on 7 out of the 11 MF terms.

**Table 7:** AUC scores for MF terms

Algorithm	Threshold	Top n	Weighted threshold	TermAUC
Prior	0.867	0.742	0.795	0.500
BLAST	0.794	0.779	0.734	0.634
Gotcha	0.786	0.774	0.728	0.665
*k*NN (1 source)	0.814	0.780	0.747	**0.702**
MS*-k*NN (3 sources)	**0.883**	**0.784**	**0.819**	0.701

**Table 8:** AUC scores for BP terms

Algorithm	Threshold	Top n	Weighted threshold	TermAUC
Prior	**0.898**	0.630	**0.822**	0.500
BLAST	0.771	0.633	0.697	0.648
Gotcha	0.748	0.637	0.677	**0.666**
*k*NN (1 source)	0.811	**0.642**	0.724	0.651
MS-*k*NN (3 sources)	0.893	0.636	0.818	0.650

#### Post-CAFA analysis

We performed several additional experiments to get a better insight into the proposed algorithms and to explore some alternatives. Among the 595 test proteins, 66 proteins were in 2 data sources, and only 10 proteins in all 3 data sources. Among these 10 proteins, 8 of them were annotated with MF terms and 8 of them with BP terms. We studied results on these 10 proteins in more detail. While the results in Tables 9 and 10 should not be interpreted in terms of statistical significance due to small sample size (for that, we point a reader to Table 5), they provide an insight into improved accuracy of MS-*k*NN as compared to similarity-based *k*NN on 595 CAFA proteins. Because we had only 8 proteins for evaluation for both MF terms and BP terms, the TermAUC accuracies for GO terms were not reliable and are not shown. As can be seen, AUC of MS-*k*NN was much larger than that of similarity-based *k*NN on all accuracies except Top n AUC on BP terms. We note that these results are consistent with those reported in Table 5, that were obtained on 1,302 test proteins.

**Table 9:** Comparison of AUC scores on 8 test proteins based on MF terms

Algorithm	Threshold	Top n	Weighted threshold
*k*NN (1 source)	0.853	0.740	0.768
MS-*k*NN (3 sources)	0.949	0.845	0.910

**Table 10:** Comparison of AUC scores on 8 test proteins based on BP terms

Algorithm	Threshold	Top n	Weighted threshold
*k*NN (1 source)	0.798	0.526	0.696
MS-*k*NN (3 sources)	0.920	0.526	0.846

## Conclusions

The protein function prediction is a complex problem. In this paper, we focused on the question of how to integrate multiple data sources to improve the prediction accuracy. We discussed and evaluated several different integration schemes in this paper. Our pre-CAFA and CAFA results strongly indicate that integrating information from multiple data sources could improve protein function prediction accuracy. At the level of sequence similarity-based predictions, we observed that it is beneficial to consider all available annotated proteins, regardless how evolutionary distant they are from a query protein. Considering the time limitations associated with the tight deadline for submission of the CAFA predictions, our strategy to use the simple and efficient *k*-NN algorithm, coupled with simple integration of prediction scores from multiple data sources, proved to be reasonable.

There are certainly many avenues for future improvements of function predictions. A straightforward one is to include as many available sources of structural and functional protein information. For example, in CAFA, we used only microarray data from a single, albeit commonly used, human microarray platform. Information beyond microarray data and protein-protein interaction data, such as chromosomal neighborhood of a gene, mutations, role in various diseases, or protein structure, could certainly be valuable. Based on our experience during CAFA, we think that further advances in statistical approaches for function prediction are needed. Particularly, we would like to point to two open problems we believe could lead to significant advances in protein function prediction. One is related with the observation that a lack of a protein's annotation with a certain GO term should not be treated as negative evidence, but rather as a missing label. As a consequence, it would be advisable to treat function prediction problem as a one class classification, instead of binary classification. Another is the problem of sampling bias, created by the fact that available annotations are not a random sample from the protein/term space. Developing methods that are robust to sampling bias or the ones that could correct its negative effects should be one of the priorities of future research in the field.

## Methods

Below, we describe *k*-nearest neighbor (*k*NN) classifiers we evaluated and used during CAFA, as well as the approaches for integration of predictions from multiple data sources.

### Baseline *k*NN classifier

To calculate a likelihood that protein *p *has function *f*, we used a weighted variant of the *k*NN algorithm, as proposed in (Pandey et al., 2009) [15]. The prediction score of function *f *for protein *p *is calculated as

(1) $$score\left(p,f\right)=\underset{p^{\prime }\in N_{k}\left(p\right)}{\sum }sim\left(p,p^{\prime }\right)I\left(f\in functions\left(p^{\prime }\right)\right),$$

where *sim*(*p, p*') denotes the similarity score between proteins *p *and *p*', *I *is an indicator function that returns 1 if *p*' is experimentally annotated with *f *and 0 otherwise, and *N_k_*(*p*) is the set of the *k *nearest neighbors of *p *according to the metric *sim*. The similarity score between two proteins *sim*(*p, p*') on each of the three data sources we considered was calculated in the following way. For protein sequence data, the similarity score was calculated as percent identity divided by 100. For microarray data, Pearson correlation coefficient, a popular method for measuring the similarity between gene expressions [16], was used as the similarity score between two proteins. For protein-protein interaction data, the similarity score was set to 1 if two proteins interacted and 0 otherwise. We note that more sophisticated similarity score could be used by considering reliability of PPI information, but we did not pursue it due to the CAFA time limitations.

### Lin-sim *k*NN classifier

In protein function prediction problem, a single protein may have multiple functions, and the functions are organized in a hierarchy. Pandey et al [15] proposed a method that incorporates contributions not only from the neighboring proteins annotated with the same function, but also from proteins annotated with *similar *functions. Their proposed prediction model is

(2) $$\begin{array}{c} score\left(p,f\right)=\underset{p^{\prime }\in N_{k}\left(p\right)}{\sum }sim\left(p,p^{\prime }\right)\left(\underset{f^{\prime }\in functions\left(p^{\prime }\right)}{\sum }linsim\left(f^{\prime },f\right)\right), \end{array}$$

where *linsim*(*f*', *f*) denotes the similarity score between functions *f *and *f*'. The Lin's similarity measure [17] is used to compute the similarity between two concepts in a hierarchy. It is calculated as

(3) $$linsim\left(f,f^{\prime }\right)=\frac{2\times \left[\text{log}p_{ms}\left(f,f^{\prime }\right)\right]}{\text{log}p\left(f\right)+\text{log}p\left(f^{\prime }\right)},$$

where *f *and *f*' are the Gene Ontology terms. The *p*(*f*) denotes the probability of a protein being annotated with function *f*, which is estimated from the available set of GO annotations. The joint probability *p_ms _*(*f, f*') is calculated as $p_{ms}\left(f,f^{\prime }\right)=\underset{l\in S\left(f,f^{\prime }\right)}{\text{min}}p\left(l\right)$, where *S*(*f, f*') is a set of common ancestors of functions *f *and *f*'. It is easy to see that *linsim*(*f, f*') = 1 when *f *= *f*', and *limsim*(*f, f*') = 0 when their minimum subsume is the root of the ontology. Thus, the *limsim *score is always in the 0[1] range.

### Integration of scores from multiple data sources

By using equations (1) or (2), we can obtain scores for each protein/function pair (*p, f*). In particular, we can obtain one score using sequence similarity and one using PPI. Since we used *J *(= 392) microarray data sets, we had one prediction score for each gene expression data set. Given the *J *+ 2 scores for each (*p, f*) pair, an open question was what is the best way to integrate them into a final, single score. We studied the following prediction score integration schemes: (1) averaging; (2) the same weighted averaging for any (*p, f*) pair; (3) different weighted averaging for different GO term clusters. In the schemes (2) and (3), the weights for different data sources were obtained by solving a convex optimization problem.

### Averaging (MS-*k*NN)

Let us denote by *score^SEQ^*(*p, f*) the score obtained from sequence similarity data, by *score^PPI^*(*p, f*) the score obtained from PPI data, and by *scorej^EXP^*(*p, f*) the score obtained by the *j*-th microarray data set. By averaging, the final score is obtained as

(4) $$score\left(p,f\right)=\frac{1}{3}score^{SEQ}\left(p,f\right)+\frac{1}{3}score^{PPI}\left(p,f\right)+\frac{1}{3J}\overset{J}{\underset{j=1}{\sum }}score_{j}^{EXP}\left(p,f\right).$$

We call the resulting algorithm the *Multi-Source kNN *(MS-*k*NN), as this was the final algorithm we used in CAFA.

### Weighted averaging (MS-**w**-*k*NN)

MS-*k*NN assumes that each data source is equally informative, which might not hold in general. We thus considered using weighted averaging of the scores from different sources. For MS-**w**-*k*NN we learned the weights from training data using a large margin method as follows. Let us assume that we are given *m *data sources, {*Dj, j *= 1..*m*}, and *n *proteins {*x_i_, i *= 1..*n*}. Each protein is assigned to several functions from the set of *k *functions. Let *Y_i _*denote the set of functions that protein *x_i _*is assigned to, and ${Ȳ}_{i}$ the set of functions that protein *x_i _*is not assigned to. Furthermore, let *f*(*x, y*) be a vector of length *m*, whose *j*-th element is the score of protein *x *for function *y *on the data source *D_j_*. Then, a weight vector **w**, used for averaging of *m *prediction, is found by minimizing the following optimization problem,

(5) $$\begin{array}{l} \underset{W,\xi }{\text{min}}\underset{i}{\sum }\underset{y\in Y_{i},ȳ\in Ȳi}{\sum }\xi_{i}\left(y,ȳ\right)\\ s.t\;W^{T}\left(f\left(x_{i},y\right)-f\left(x_{i},ȳ\right)\right)\geq -\xi_{i}\left(y,ȳ\right),\forall i,y\in Y_{i},ȳ\in {Ȳ}_{i}\\ \xi_{i}\left(y,ȳ\right)\geq 0,\forall i,y\in Y_{i},ȳ\in {Ȳ}_{i}\\ W^{T}e=1;W\geq 0 \end{array}$$

where **e **is a vector of ones. The resulting convex optimization problem can be solved using standard optimization tools, such as CVX (http://cvxr.com/cvx/). With the trained weight vector **w**, the protein-function scores from different data sources can be integrated by taking their weighted average as **w**^T^·*f*(*x, y*).

### Cluster-specific weighted averaging (MS-CW-*k*NN)

Instead of learning a single weight for all GO terms, we can partition functions into clusters and assign cluster specific weights. Since the Gene Ontology is already organized in a hierarchical structure, we can directly use it to cluster the GO terms. In MS-CW-*k*NN, we considered clustering of all GO terms at the root level to MF and BP terms, and at the first level to 7 MF and 25 BP functional clusters.

## Competing interests

The authors declare that they have no competing interests.

## Authors' contributions

LL, ND, YG and SV conceived the study and developed the evaluation strategies. SV and YG directed the work. LL and ND implemented all methods and performed the experiments. LL wrote the first draft of the manuscript. All authors participated in the preparation of the manuscript and approved the final version.

## Declarations

This article has been published as part of *BMC Bioinformatics *Volume 14 Supplement 3, 2013: Proceedings of Automated Function Prediction SIG 2011 featuring the CAFA Challenge: Critical Assessment of Function Annotations. The full contents of the supplement are available online at URL. http://www.biomedcentral.com/bmcbioinformatics/supplements/14/S3.
